# Tropical protected areas reduced deforestation carbon emissions by one third from 2000–2012

**DOI:** 10.1038/s41598-017-14467-w

**Published:** 2017-10-25

**Authors:** Daniel P. Bebber, Nathalie Butt

**Affiliations:** 1 Department of Biosciences, University of Exeter, Exeter, EX4 4QD UK; 2 School of Biological Sciences, University of Queensland, Brisbane, QLD 4072 Australia

## Abstract

Tropical deforestation is responsible for around one tenth of total anthropogenic carbon emissions, and tropical protected areas (PAs) that reduce deforestation can therefore play an important role in mitigating climate change and protecting biodiversity and ecosystem services. While the effectiveness of PAs in reducing deforestation has been estimated, the impact on global carbon emissions remains unquantified. Here we show that tropical PAs overall reduced deforestation carbon emissions by 4.88 Pg, or around 29%, between 2000 and 2012, when compared to expected rates of deforestation controlling for spatial variation in deforestation pressure. The largest contribution was from the tropical Americas (368.8 TgC y^−1^), followed by Asia (25.0 TgC y^−1^) and Africa (12.7 TgC y^−1^). Variation in PA effectiveness is largely driven by local factors affecting individual PAs, rather than designations assigned by governments.

## Introduction

Tropical forests account for around 68% of global forest carbon stocks, in terms of live biomass, above and below ground^1,2^. Carbon sequestration and storage in terrestrial systems is an important mechanism for climate change mitigation^3^, and deforestation and land clearing are significant drivers of carbon emissions in tropical systems, as deforestation is the most important contributor to emissions from land use change^4^. Between 2000 and 2010, tropical deforestation and land use change emitted 1.0 PgC y^−1^ net to the atmosphere^5,6^. When forest degradation is also included^7^, tropical emissions between 1990 and 2010 were around 1.4 PgC y^−1^.

Protected areas (PAs) comprise up to 15% of the land surface globally across all ecosystems^8–10^, and are intended to protect habitats^11^ as well as benefit local communities through ecosystem service provision and the support of sustainable livelihoods^12^. Although PAs can achieve conservation goals^13,14^, deforestation does occur within PAs^9^; in some cases increasing human pressure (hunting and timber extraction nearby) is driving biodiversity loss and decline^11^. While it may be difficult to achieve both biodiversity and socio-economic benefits, there is evidence that where people are included as stakeholders, mutually beneficial scenarios are achievable^14,15^. PAs vary widely in extent and type, from local ecological or indigenous reserves through to national parks and World Heritage Sites. The International Union for Conservation of Nature (IUCN) categorization system comprises six broad classes, relating to their management objectives^16,17^: Category Ia denotes strict nature reserves, Ib wilderness areas without significant human habitation, II national parks set aside to control large-scale ecological processes, III natural monuments or feature likely to have high visitor value, IV habitats or species management areas often requiring management intervention, V protected landscapes or seascapes featuring significant human influence, and VI areas with sustainable use of natural resources. The ability of category V and VI to both conserve biodiversity and promote economic welfare remains contested^17^.

Previous tropical or global analyses of the effectiveness of PAs have focused on biodiversity conservation, forest loss or social outcomes^8,9,18,19^, but there has been little analysis of the impacts on carbon stocks and losses^20,21^, which influence climate change-related biophysical feedbacks^22^. While total soil carbon content in forests is 2-3 times greater than that stored in above ground biomass (AGB)^5^, it is not always readily released to the atmosphere after deforestation; whether it is or not depends on the post-deforestation land use^5^. Root, or belowground, biomass (BGB) is generally equivalent to one-fifth of the AGB in tropical forests^23^: here we focus on AGB, in line with other recent studies^3^. Recent high-resolution forest cover^24^ and above ground biomass (AGB)^5^ estimates allow large-scale analysis of PAs in terms of reduction of forest canopy loss and carbon emissions. We analysed the effectiveness of PAs in reducing deforestation and forest carbon loss, investigating variation among PA designations and controlling for spatial variation in deforestation pressure. Using empirical relationships between canopy cover and AGB, we estimated the influence of PAs on tropical forest carbon emissions resulting from reductions in deforestation rates.

Deforestation rates within PA borders are significantly lower than outside^25,26^, and they are especially important for forest conservation in developing countries^27^. However, the siting of PAs is not random, and they are often located in areas that are inaccessible or unsuitable for agriculture, far from cities or transport links, and in topographically challenging areas (i.e., on steep slopes), such that they are unlikely to be under pressure from the developmental drivers of land use change^8^. Because of non-random PA locations, and because deforestation rate varies among countries due to political and socioeconomic factors^9^, PA effectiveness may be overestimated^28^, and so statistical models of forest loss in unprotected (non-PA) areas are commonly used to control for this bias^18,20,29^. We analysed the difference (*r*_*d*_) between observed and expected remaining forest cover in PAs to control for any biases in PA location. The expected remaining cover was determined from Generalized Additive Models (GAMs) of forest loss in non-PA regions. Positive *r*_*d*_ therefore indicates that remaining forest cover in PAs is greater than expected for a particular location, and negative *r*_*d*_ indicates that remaining forest cover is less than expected.

## Results and Discussion

We analysed the three major tropical regions, the Americas, Africa, and Asia, separately (Supplementary Table S1). Remaining canopy cover in 2012 in non-PA areas increased with the fraction of steep slopes in all three regions, but the relationships with other predictors were complex (Supplementary Fig. S1). For example, while remaining cover tended to decline with increasing road density, it increased with very high road density in Asia, perhaps because of canopy maintenance in urban areas. Remaining canopy cover tended to increase with the fraction of steep slopes, but followed non-linear relationships with population density, road density, altitude, and agricultural suitability (Supplementary Fig. S1). Controlling for non-random PA location was most important for areas with high forest cover (Supplementary Fig. S2). The fraction of variance in remaining canopy cover in non-PA areas explained by the GAM for the Americas was 95.2%, for Africa 99.0%, and for Asia 92.5%.

**Figure 1:**
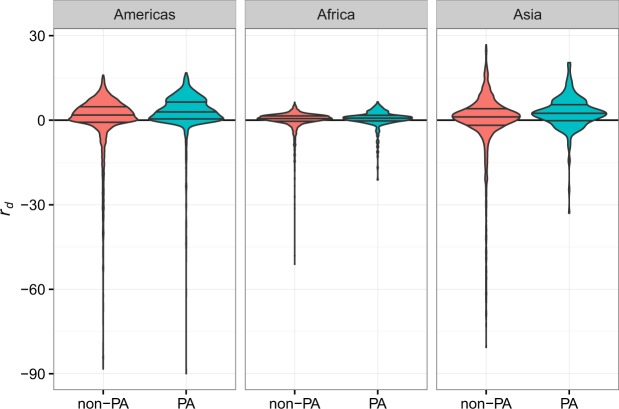
The difference between observed and expected remaining forest canopy cover, *r*_*d*_, in non-PA and PA forests. Violin-plot width is proportional to 1 arc minute pixel frequency. Horizontal lines show interquartile ranges and medians. Values for pixels with original (year 2000) canopy cover >20% are shown. Values above the zero line indicate that more canopy remained than expected by a model of remaining canopy cover in non-PA areas.

**Figure 2:**
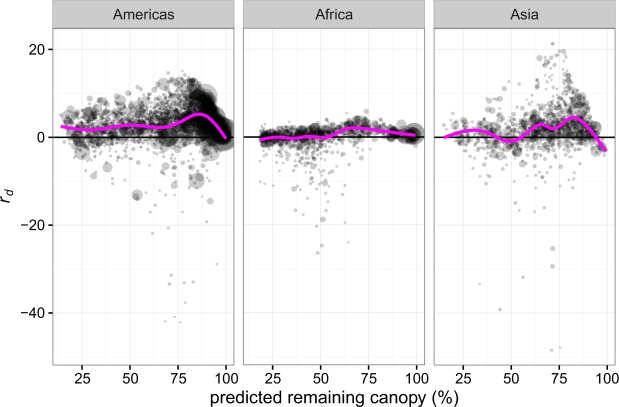
The difference between observed and expected remaining canopy cover, *r*_*d*_, against predicted remaining canopy cover in PAs for the three tropical forest regions. Shaded circles represent individual PAs with size proportional to geographic area. The magenta lines are smooths, showing the average for different predicted remaining values. PAs in the Americas have positive *r*_*d*_ across the range of predicted remaining canopy cover, while the African and Asian PAs are most effective at 60–80% of predicted remaining cover. PAs with areas <1000 ha are not plotted, because of their extremely small size at this scale.

Forest within PAs retained more canopy cover between 2000 and 2012 than would be expected for non-protected forest with similar levels of proxy variables for deforestation pressure (Fig. 1; Supplementary Table S2). Mean *r*_*d*_ per PA, weighted by PA area and controlling for spatial autocorrelation, was 2.65 ± 0.24% for the Americas (test vs. zero, t = 11.2, p < 0.001), 0.39 ± 0.10% for Africa (test vs. zero, t = 6.9, p < 0.001), and 1.92 ± 0.17% for Asia (test vs. zero, t = 11.3, p < 0.001) (Fig. 2). Mean *r*_*d*_ for all forested non-PA pixels was 0.057 ± 0.058% (spatial block bootstrap test vs. zero, Z = 0.99, p = 0.32) for the Americas, 0.004 ± 0.026% (Z = 0.16, p = 0.88) for Africa, and 0.027 ± 0.061% (Z = 0.45, p = 0.66) for Asia, confirming unbiased estimation of deforestation by the GAMs. PAs in the Americas have positive *r*_*d*_ across the range of predicted remaining canopy cover, while the African and Asian PAs are most effective at 60–80% of predicted remaining cover (Fig. 2).

Mean *r*_*d*_ for PAs varied significantly among countries within all three tropical regions, confirming that reserve effectiveness is partly determined by governance, but the influence of other predictors was variable (Supplementary Table S2; Supplementary Fig. S3). There was a strong influence of spatial location, in addition to significant variation among countries. *r*_*d*_ varied significantly with PA original canopy cover in all regions, increasing overall, while *r*_*d*_ decreased at high PA area in Africa and Asia. For status year, *r*_*d*_ decreased slightly for recently-gazetted PAs in the Americas, with no significant effect in Africa and Asia (Supplementary Table S2; Supplementary Fig. S3). We predicted the difference among designations and IUCN categories for an example set of PAs from one country in each region (Supplementary Fig. S4). In Brazil, Ecological Station and Biological Reserve were associated with greatest protection, while Environmental Protection Area had the least protection. In Ghana, there was little variation among designations. In Indonesia, RAMSAR Site is associated with the greatest protection. In Brazil, IUCN Class IV had the most positive influence on *r*_*d*_, but there was little significant variation among the other categories, nor among categories in the other tropical regions (Supplementary Figs S5 and S6). This contrasts with an earlier finding that strict protection (i.e. IUCN Category 1a) provides the greatest protection^18^. While statistically significant, the influence of designations and IUCN Categories was not easily interpretable, and varied among the tropical regions, while a large fraction of the variance in *r*_*d*_ among PAs remained unexplained by our large-scale models, particularly in Africa (Supplementary Table S2). The particular socioeconomic conditions within and around individuals PAs would need to be evaluated to fully understand why protection is achieved in some cases, while in other cases it fails^13,30^.

**Figure 3:**
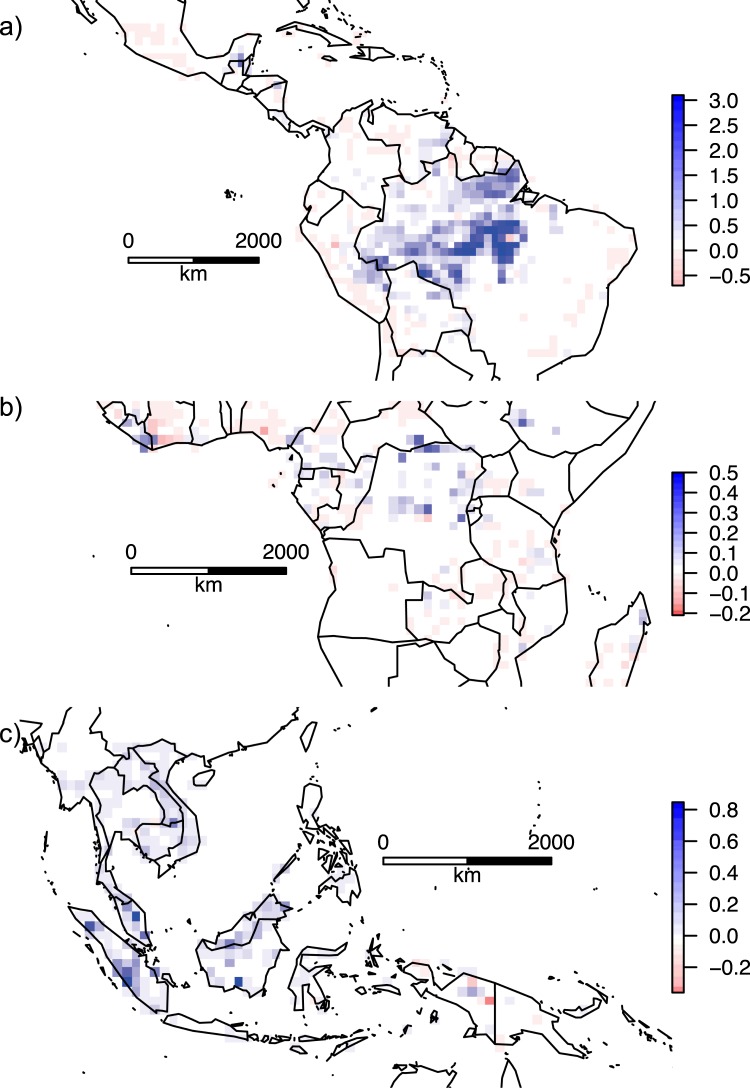
Annual estimated carbon saving in tropical PAs (Mg C ha^−1^ y^−1^) from 2000–2012, aggregated to 1 degree pixels, in (**a**) Americas, (**b**) Africa, (**c**) Asia. Red hues indicate carbon loss greater than expected for non-PA areas, blue hues indicate carbon retention greater than expected. This does not include changes in forest carbon in unprotected areas. Images were created using *R* v. 3.4.0^38^. Specifically, rasters were created with package *raster* v.2.5.8, and country boundaries drawn using package *maps* v.3.2.0.

Tropical forest AGB estimates^5^ were strongly related to canopy cover, and based upon empirical relationships (Supplementary Fig. S6) we found that PAs lying within tropics in the Americas retained 4.42 Pg C more than expected for non-PA areas between 2000 and 2012, equivalent to 368.8 Tg C y^−1^. Brazilian PAs contributed 82% of this total (Supplementary Table S4). African PAs retained 0.15 Pg, the equivalent of 12.7 Tg C y^−1^ more than expected, and Asian PAs, 0.3 Pg total, or 25.0 Tg C y^−1^ (Fig. 3). Tropical American PAs covered nearly five times more forest area (>20% canopy cover) than tropical Africa PAs, and nearly seven times more than the area of tropical Asian PAs, contributing to the much larger carbon saving in the Americas. Though analyses of deforestation rates in PAs are common, estimates of carbon emissions reductions are rare^21^. We estimated total annualized AGB carbon losses of 0.79 Pg C y^−1^ for unprotected areas and 0.096 Pg C y^−1^ for PAs across the tropics (Supplementary Table S1), compared with 0.62 Pg C y^−1^ and 0.054 Pg C y^−1^ estimated in a previous study using different source data for deforestation and carbon density^21^. The relative contributions of PAs to emissions reductions differs substantially between countries. In our analysis, Brazilian PAs contributed 25 times the benefit of Indonesian PAs, compared with five times the benefit in a previous study based on older data^20^. One reason for this difference could be the decline in deforestation rates in Brazil, which have almost halved since 2008, and increased in Indonesia since 2008 ref.^24^.

Our estimate of the effectiveness of PAs in retaining forest cover and carbon, quantified as *r*_*d*_, represents an upper limit because of the potential for leakage, or displacement, of deforestation outside PA boundaries^31^. We tested for an effect of local leakage within a few kilometres of PA boundaries on our estimates of deforestation pressure, and found none. However, this does not preclude the possibility that larger-scale displacement of deforestation occurs. The problem of leakage, either locally or internationally, has been the subject of intense debate in the development of REDD + processes during international climate change mitigation negotiations^32^. In the worst case, all deforestation that would have occurred inside the PAs we analysed could have been displaced to outside the PA boundaries, and the benefit of the PAs would be zero. The implications for conservation could be even more negative if the displaced deforestation occurs in more vulnerable habitats and ecosystems than those inside the PAs. While local leakage is variable^33^, and appears to be unimportant^20^, large scale leakage remains entirely unquantified in recently published studies of multi-national REDD+ projects^34^. Until more is known of the importance of leakage under different geographical and social situations, we are limited to reporting the upper range of PA carbon benefits.

The Paris Agreement resulting from the United Nations Framework Convention on Climate Change Conference of Parties (UNFCCC COP) 21 included reduction of deforestation as a key activity in mitigating climate change to 2020, and our analysis suggests that tropical PAs have played a significant role in conserving forest carbon in recent years, equivalent to a reduction of around 29% of tropical deforestation emissions. Global carbon sinks will be critical for meeting the Paris Agreement, and tropical deforestation risks rainforest carbon sinks switching to carbon sources^35^. The potential additionality^32^ of PAs is greatest in countries with the highest background deforestation rates and forests with the highest carbon densities, and support of these should be prioritized. In many of these countries, intact forest landscapes, important for carbon storage across large areas have been reduced and should be prioritised for inclusion in PAs. Carbon storage, along with socioeconomic and biodiversity benefits, provides further support for the need to maintain the world’s protected area network.

## Methods

In summary, our analysis proceeded in five stages: first, we estimated observed remaining canopy cover in 2012 (*r*_*obs*_) in all forest areas, using data on forest cover in 2000 and loss by 2012. Second, we constructed statistical models of *r*_*obs*_ in unprotected areas using a number of land-use and environmental predictors. Third, we predicted the expected remaining canopy cover (*r*_*exp*_) in PAs, from predictor variables within PAs using the models. Fourth, we calculated the difference between observed and expected canopy cover as *r*_*d*_ = *r*_*obs*_−*r*_*exp*_, to give a measure of PA effectiveness relative to background deforestation pressure. Fifth, we estimated empirical relationships between above-ground forest carbon density and canopy cover, and used these relationships to estimate the difference between observed and expected remaining carbon within PAs.

Full descriptions of all datasets are given in the Supplementary Information. We estimated the mean remaining canopy cover from original canopy cover in 2000 and loss by 2012, by setting those pixels reported as having lost forest to zero canopy, under the assumption that forest loss was complete for those pixels, and that forest would not have grown back by 2012. The original data were at 1 arc second (~30 m) resolution, and we aggregated these to 1 arc minute (~1.8 km) resolution by taking means. We used Generalized Additive Models (GAMs)^36^ parameterized using observed remaining canopy cover in 2012 in non-PA areas, to predict expected remaining canopy cover in PAs, and compared these predictions with the observed remaining canopy cover in PAs. The difference between observed and expected remaining canopy cover (*r*_*d*_) was used to analyse the effectiveness of PAs in reducing deforestation. Positive *r*_*d*_ values indicated that remaining cover was greater than expected for a non-PA area with the same values of predictor variables, and negative *r*_*d*_ indicated that remaining cover was lower than expected. GAMs have been previously used in analyses of PA effectiveness^29^, and are useful when the underlying relationship between predictor and response is non-linear, and is not readily defined by a particular mathematical function. We checked for bias in GAM estimates of remaining forest cover for non-PA areas by testing *r*_*d*_ vs. zero, using spatial block bootstrap resampling^37^ with data aggregated to 1° blocks, weighted by the fraction of non-PA forest pixels per block, and 1000 bootstrap samples. All analyses were conducted in R v.3.4.0.ref.^38^.

In addition to original canopy cover in 2000, we used the following predictors of remaining forest cover in 2012 in our GAMs, which have all been implicated in previous studies of tropical deforestation^8,15,29^: Country (as a fixed factor), human population density^39^, road density^40^, agricultural suitability^41^, altitude^42^ and fraction of steep slopes^42^ as the area per pixel with a slope >15°. All continuous variables were fitted as cubic regression splines (population density, road density, and steep slope fraction were log-transformed), and we included spatial location as a two-dimensional tensor product smoothed across latitude and longitude to control for other, unidentified sources of spatial variation in deforestation pressure. No other interaction terms were included, and separate models were fitted for each tropical region (Latin America, Africa and Asia). All datasets were transformed to 1 arc minute, either by aggregation to the mean value or bilinear interpolation, if the original dataset was at higher or lower resolution, respectively. The responses of remaining cover to predictors are shown in Supplementary Fig. S1. We found the largest differences at high predicted values, demonstrating the importance of controlling for non-random PA locations (Supplementary Fig. S2).

The difference between predictions from GAMs including all our deforestation pressure proxies (country, population density, road density, slope steepness, altitude, and agricultural suitability) and null model using only original canopy cover was small indicating a relatively small influence of these predictors on deforestation pressure (Fig. S3). A fraction of predictions were slightly negative (to around −5%) and were set to zero, and those exceeding 100% (to around 110%) were set to 100%. For the Americas, 8.6% of pixels were predicted to have negative remaining cover, and 4.4% to have above 100%. For Africa, 0.9% were negative and 0.4% above 100%. For Asia, 1.3% were negative, and 4.8% above 100%.

The mean difference between observed and predicted remaining canopy cover (*r*_*d*_) was calculated for each PA, omitting PAs with total areas below the minimum pixel size of 1 arc minute (approximately 300 ha). PAs above this threshold represented 97%, 94% and 90% of the total PA area in the Americas, Africa and Asia, respectively. We then fitted GAMs to PA mean *r*_*d*_ weighted by PA area, such that larger PAs were more influential on the fit (Supplementary Table S2). Categorical predictors were country, PA designation, and IUCN category, while cubic splines were fitted for PA area, status year (year that the PA was gazetted), original canopy cover, and the fraction of the PA area covered by other PAs (the area of some PAs were partially covered by other PAs). Mean *r*_*d*_ per PA for each region was estimated using generalized least squares models, weighted by PA area and controlling for spatial autocorrelation using a spherical autocorrelation model^43^.

We estimated carbon loss from deforestation using 15 arc second resolution aboveground live woody biomass (AGB) data^5^. Biomass data were estimated from 2007-8 imagery and hence were not contemporaneous with our estimates of canopy cover (2000 and 2012). To estimate biomass in 2000 and 2012 we derived models of biomass from canopy cover and ecosystem type, for only those pixels that did not change canopy cover by more than 1% between 2000 and 2012. We aggregated biomass to 1 arc minute resolution, and fitted a smoothing spline using canopy cover as the predictor for each tropical region (Supplementary Fig. S6). We were unable to use canopy height as a predictor of AGB in 2000 because global canopy height databases^44^ are only available post-2000. We compared these univariate fits with splines that varied by ecosystem (see Supplementary Material for description of ecosystem data), using GAMs. Inclusion of ecosystem type improved the model R^2^ from 92.7 to 93.4% for the Americas, from 90.0 to 90.5% for Africa, and from 90.8 to 91.8% for Asia. These improvements in model fit were trivial and so we estimated original and remaining biomass from original and remaining canopy cover using the simple models, omitting the minor variations due to ecosystem class. We then estimated AGB from the predicted remaining canopy cover for PAs, using the remaining canopy cover model parameterized using non-PA areas. AGB per unit area was converted to above-ground carbon by multiplying the AGB by pixel area in hectares, and using a factor of 0.5 to convert from biomass to carbon^45^. The difference between the remaining AGB estimates and the predicted remaining AGB estimates for pixels within PAs was summed to give the overall carbon saving per region. Only pixels with original canopy cover greater than 20%, and within the tropics (between 23°N and 23°S) were included in the carbon saving calculation.

Leakage, the displacement of deforestation from inside to outside PAs, could bias the results by spuriously elevating the observed deforestation rate in non-PA regions^20,32^. We tested for an effect of local leakage by re-fitting GAMs for remaining forest cover in 2012, but excluding non-PA areas in a buffer around all PAs^20^. We chose buffer widths of similar magnitude to those used in a previous study^20^, but which increased with PA size such that buffer depth in kilometers equalled the natural logarithm of PA area in square kilometres (truncated to zero below 1 km^2^ area), as the potential for displacement of deforestation range of 5 km for the majority of PAs, used in the previous study, with a maximum around 10 km for the largest PAs (Supplementary Fig. S7). Predictions for remaining forest cover within PAs, estimated from GAMs fitted to non-PA regions excluding buffers, were almost identical to those estimated from GAMs fitted to all non-PA forest areas. Spearman correlation coefficients were 0.99996 for the Americas, 0.99995 for Africa, and 0.9987 for Asia. Hence, we concur with earlier studies that short-range leakage is undetectable and can be ignored in analysis, and that longer-range leakage, where deforestation is displaced widely (perhaps internationally), is intractable^20^.

## Electronic supplementary material

Supplementary Information

## References

[1] Pan Y (2011). A Large and Persistent Carbon Sink in the World’s Forests. Science.

[2] Saatchi SS (2011). Benchmark map of forest carbon stocks in tropical regions across three continents. Proc. Natl. Acad. Sci..

[3] Houghton RA, Byers B, Nassikas AA (2015). A role for tropical forests in stabilizing atmospheric CO2. Nat. Clim. Change.

[4] Le Quéré C (2016). Global Carbon Budget 2016. Earth Syst. Sci. Data.

[5] Baccini A (2012). Estimated carbon dioxide emissions from tropical deforestation improved by carbon-density maps. Nat. Clim. Change.

[6] Harris NL (2012). Baseline Map of Carbon Emissions from Deforestation in Tropical Regions. Science.

[7] Houghton RA (2013). The emissions of carbon from deforestation and degradation in the tropics: past trends and future potential. Carbon Manag..

[8] Joppa LN, Pfaff A (2009). High and Far: Biases in the Location of Protected Areas. PLoS ONE.

[9] Heino M (2015). Forest Loss in Protected Areas and Intact Forest Landscapes: A Global Analysis. PLoS ONE.

[10] UNEP-WCMC & IUCN. Protected Planet Report 2016. 73 (UNEP-WCMC & IUCN, 2016).

[11] Laurance WF (2012). Averting biodiversity collapse in tropical forest protected areas. Nature.

[12] Oldekop JA, Holmes G, Harris WE, Evans KL (2015). A global assessment of the social and conservation outcomes of protected areas. Conserv. Biol..

[13] Coetzee BWT, Gaston KJ, Chown SL (2014). Local Scale Comparisons of Biodiversity as a Test for Global Protected Area Ecological Performance: A Meta-Analysis. PLoS ONE.

[14] Geldmann J (2013). Effectiveness of terrestrial protected areas in reducing habitat loss and population declines. Biol. Conserv..

[15] Nelson A, Chomitz KM (2011). Effectiveness of Strict vs. Multiple Use Protected Areas in Reducing Tropical Forest Fires: A Global Analysis Using Matching Methods. PLOS ONE.

[16] Dudley, N. *Guidelines for Applying Protected Area Management Categories*. (IUCN, 2008).

[17] Shafer CL (2015). Cautionary thoughts on IUCN protected area management categories V–VI. Glob. Ecol. Conserv..

[18] Nolte C, Agrawal A, Silvius KM, Soares-Filho BS (2013). Governance regime and location influence avoided deforestation success of protected areas in the Brazilian Amazon. Proc. Natl. Acad. Sci..

[19] Bowker JN, De Vos A, Ament JM, Cumming GS (2017). Effectiveness of Africa’s tropical protected areas for maintaining forest cover. Conserv. Biol..

[20] Ferraro PJ (2015). Estimating the impacts of conservation on ecosystem services and poverty by integrating modeling and evaluation. Proc. Natl. Acad. Sci..

[21] Scharlemann JPW (2010). Securing tropical forest carbon: the contribution of protected areas to REDD. Oryx.

[22] Bonan GB (2008). Forests and climate change: forcings, feedbacks, and the climate benefits of forests. Science.

[23] Reich PB (2014). Temperature drives global patterns in forest biomass distribution in leaves, stems, and roots. Proc. Natl. Acad. Sci..

[24] Hansen MC (2013). High-Resolution Global Maps of 21st-Century Forest Cover Change. Science.

[25] Leverington F, Costa KL, Pavese H, Lisle A, Hockings M (2010). A Global Analysis of Protected Area Management Effectiveness. Environ. Manage..

[26] Nagendra H (2008). Do Parks Work? Impact of Protected Areas on Land Cover Clearing. AMBIO J. Hum. Environ..

[27] Miranda JJ, Corral L, Blackman A, Asner G, Lima E (2016). Effects of Protected Areas on Forest Cover Change and Local Communities: Evidence from the Peruvian Amazon. World Dev..

[28] Andam KS, Ferraro PJ, Pfaff A, Sanchez-Azofeifa GA, Robalino JA (2008). Measuring the effectiveness of protected area networks in reducing deforestation. Proc. Natl. Acad. Sci..

[29] Green JMH (2013). Deforestation in an African biodiversity hotspot: Extent, variation and the effectiveness of protected areas. Biol. Conserv..

[30] Pfaff A, Robalino J, Sandoval C, Herrera D (2015). Protected area types, strategies and impacts in Brazil’s Amazon: public protected area strategies do not yield a consistent ranking of protected area types by impact. Phil Trans R Soc B.

[31] Barber CP, Cochrane MA, Souza CM, Laurance WF (2014). Roads, deforestation, and the mitigating effect of protected areas in the Amazon. Biol. Conserv..

[32] Fearnside PM (2012). The theoretical battlefield: accounting for the carbon benefits of maintaining Brazil’s Amazon forest. Carbon Manag..

[33] Robalino J, Pfaff A, Villalobos L (2017). Heterogeneous Local Spillovers from Protected Areas in Costa Rica. J. Assoc. Environ. Resour. Econ..

[34] Fischer R, Hargita Y, Günter S (2016). Insights from the ground level? A content analysis review of multi-national REDD + studies since 2010. For. Policy Econ..

[35] Rockström J (2016). The world’s biggest gamble. Earths Future.

[36] Wood, S. *Generalized Additive Models: An Introduction with R*. (CRC Press, 2006).

[37] Brenning, A. Spatial cross-validation and bootstrap for the assessment of prediction rules in remote sensing: The R package sperrorest. In *2012 IEEE International Geoscience and Remote Sensing Symposium* 5372–5375 10.1109/IGARSS.2012.6352393 (2012).

[38] R Development Core Team. *R: A Language and Environment for Statistical Computing*. (R Foundation for Statistical Computing, 2017).

[39] Center for International Earth Science Information Network (CIESIN) & Centro Internacional de Agricultura Tropical (CIAT). Gridded Population of the World Version 3 (GPWv3): Population Density Grids. Available at: http://sedac.ciesin.columbia.edu/data/collection/gpw-v3 (2005).

[40] PBL. GRIP: Global Roads Inventory Project. Available at: http://geoservice.pbl.nl/geonetwork (2013).

[41] Zabel F, Putzenlechner B, Mauser W (2014). Global Agricultural Land Resources – A High Resolution Suitability Evaluation and Its Perspectives until 2100 under Climate Change Conditions. PLoS ONE.

[42] Fischer, G. *et al*. Global Agro-Ecological Zones (GAEZ v3.0). 178 (IIASA and FAO, 2012).

[43] Pinheiro, J. & Bates, D. *Mixed-Effects Models in S and S-PLUS*. (Springer Science & Business Media, 2000).

[44] Simard M, Pinto N, Fisher JB, Baccini A (2011). Mapping forest canopy height globally with spaceborne lidar. J. Geophys. Res. Biogeosciences.

[45] Chave J (2005). Tree allometry and improved estimation of carbon stocks and balance in tropical forests. Oecologia.

