# Author Correction: Tropical protected areas reduced deforestation carbon emissions by one third from 2000–2012

**DOI:** 10.1038/s41598-018-32998-8

**Published:** 2018-10-01

**Authors:** Daniel P. Bebber, Nathalie Butt

**Affiliations:** 1 Department of Biosciences, University of Exeter, Exeter, EX4 4QD UK; 2 School of Biological Sciences, University of Queensland, Brisbane, QLD 4072 Australia

Correction to: *Scientific Reports* 10.1038/s41598-017-14467-w, published online 25 October 2017

The original version of this Article contained a repeated typographical error in the Abstract and Introduction where,

‘Gg’

now reads:

‘Tg’

This has now been corrected in the PDF and HTML versions of the Article.

