# Supplementary material for: Tropical protected areas reduced deforestation carbon emissions by one third from 2000–2012
Source: Sci Rep. 2017 Oct 25;7:14005. doi: 10.1038/s41598-017-14467-w (PMC5656627; doi:10.1038/s41598-017-14467-w)
Supplement: Supplementary file 1 — Supplementary Information [file 41598_2017_14467_MOESM1_ESM.pdf]

Supplementary Information

Tropical protected areas reduced deforestation carbon emissions by one third from 2000-2012

Daniel P. Bebber & Nathalie Butt

Figures

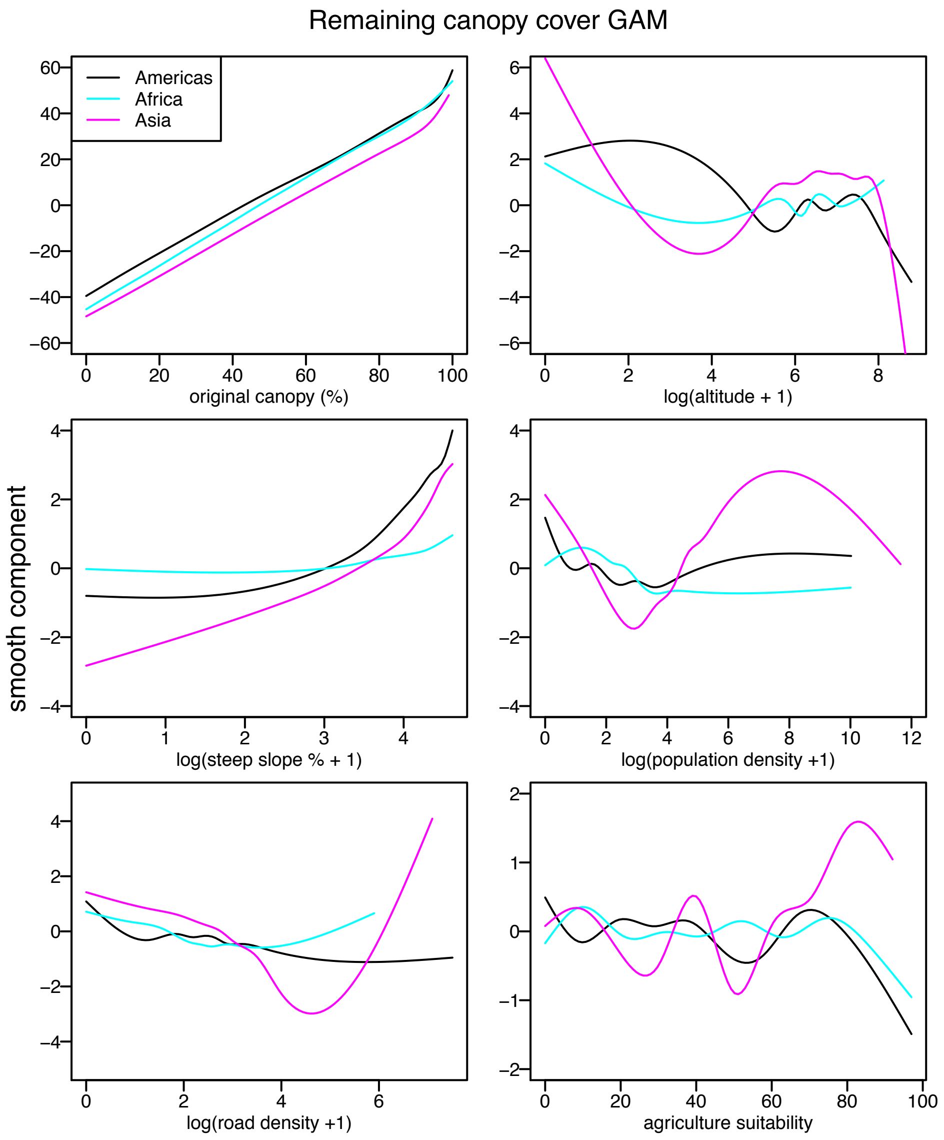

Figure S1: Smooth terms of GAMs for remaining forest cover in 2012 by tropical region. Models were fitted by 1 arc minute pixel. In addition, country was fitted as a factor, and a spatial smooth fitted to control for other large-scale variation in deforestation pressure. The smooth component is the additive contribution of the predictor to the response (i.e. remaining forest cover). Positive values indicate that remaining forest cover was greater than average, negative values indicate remaining cover was lower. Some predictors with highly skewed distributions were log-transformed to even out the distribution.

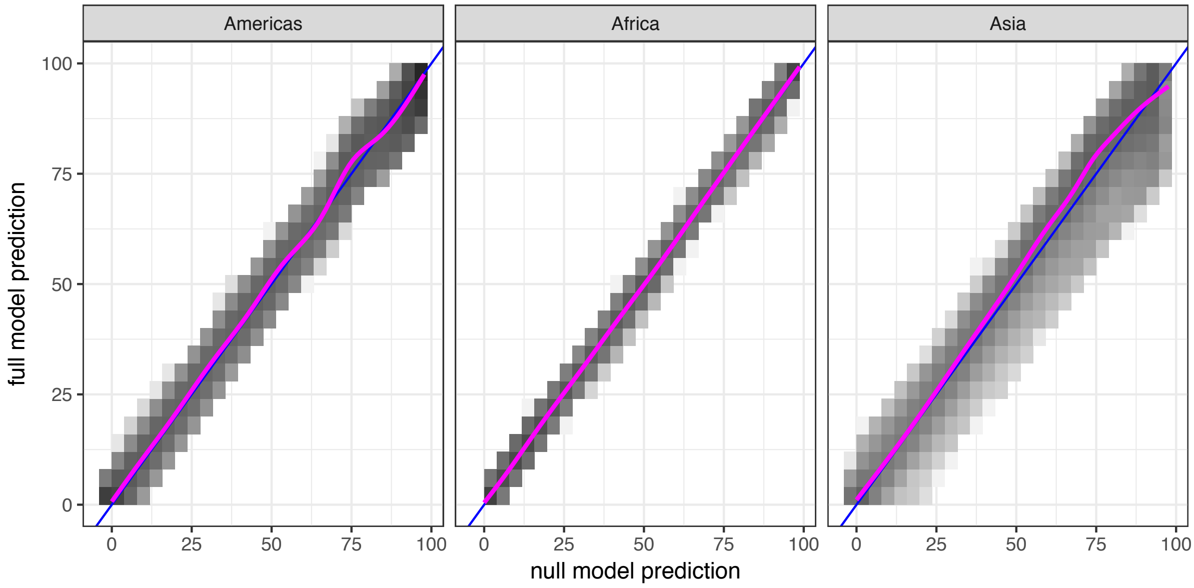

Figure S2: Comparison of GAM predictions for remaining canopy cover in PA pixels for full models (incorporating original canopy cover, country, population density, road density, altitude, fraction of steep slopes, and agricultural suitability as predictors for non-PA regions), versus predictions from a null model using only original canopy cover as a predictor. Grey shading intensity indicates the density of data. Blue sloping lines show the identity relationship, and the magenta curves show spline fits to the data. For the Americas and Asia, the full models estimated slightly greater remaining cover for PAs than the null models, at predicted remaining canopy levels of around 70-90 %. This would lead to slight overestimates of the protective effect of PAs (rd) compared with the full model accounting for variation in deforestation pressure. There was little difference between model predictions in Africa.

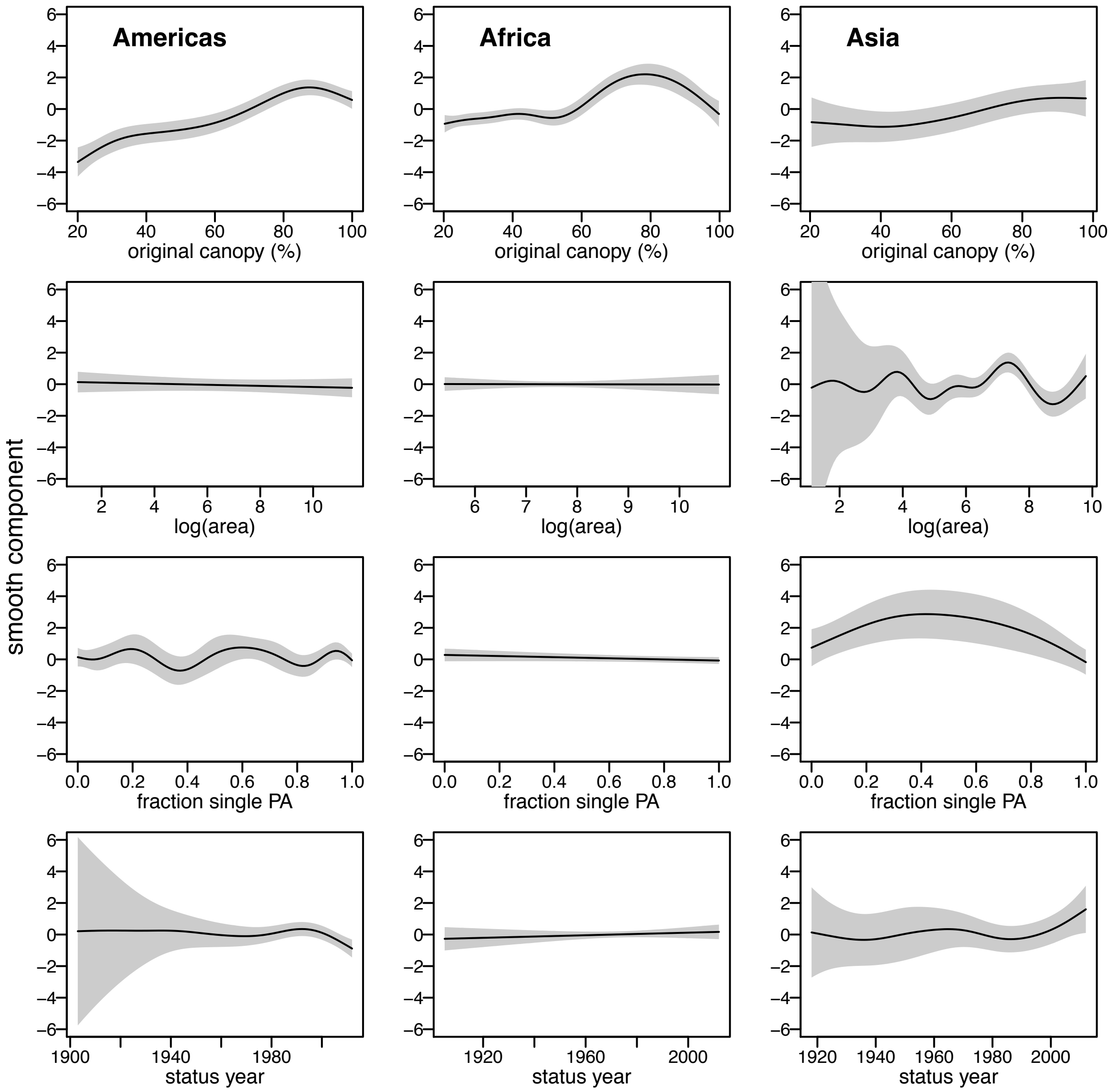

Figure S3: Smooth components for GAMs for mean r_d per PA. Smooths for each predictor are shown by row, and for each tropical region by column. Shaded areas show 95 % Confidence Limits, incorporating uncertainty around the overall mean. Only PAs with original mean forest cover >20 % and area > 300 ha were included in the model. Status year is the year in which the PA was designated. GAMs were weighted by PA area.

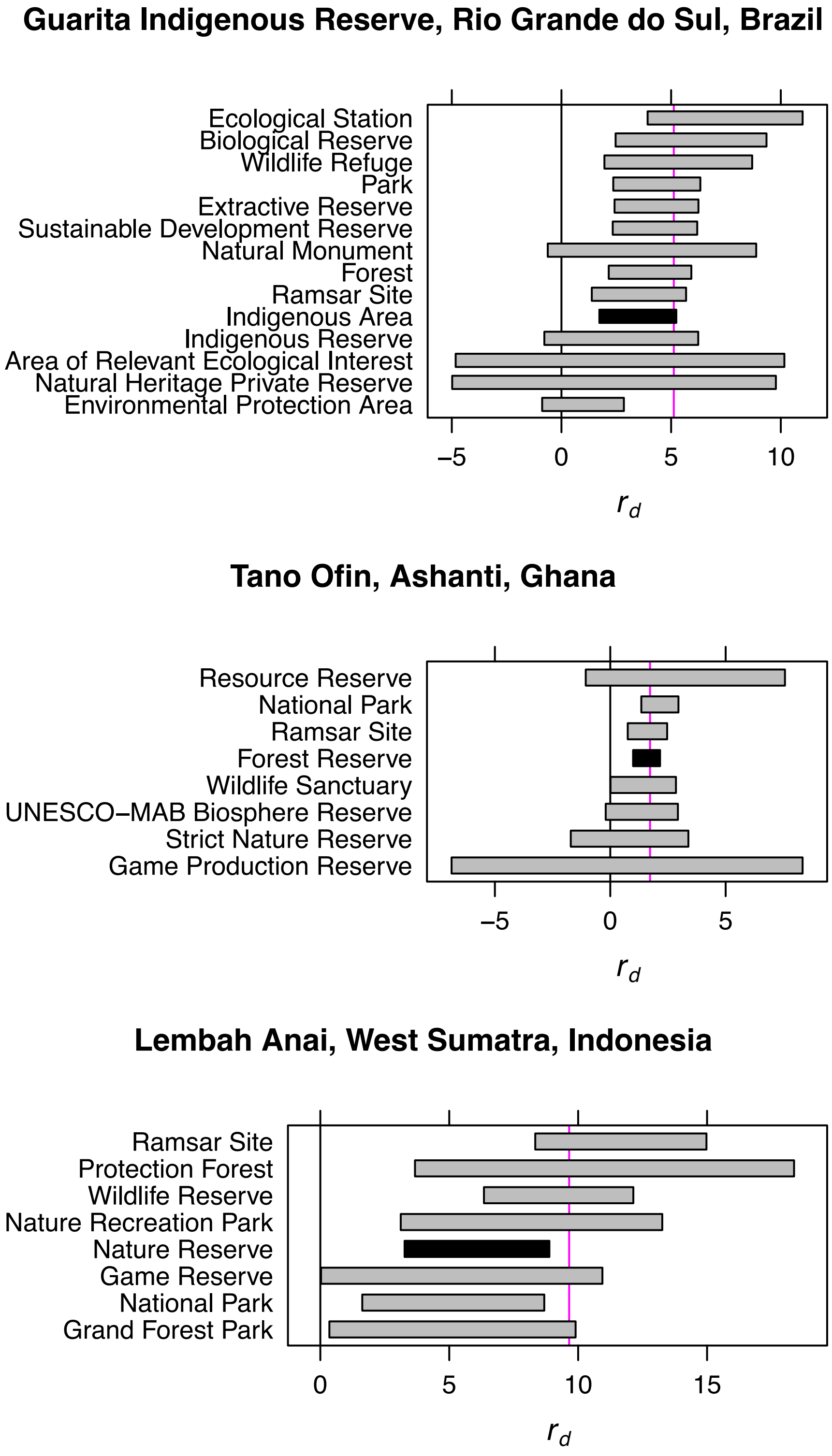

Fig. S4: Modelled effect of PA designation on r_d for an example PA in each region. Bars show 95 % Confidence Limits for predicted r_d for a hypothetical PA with properties identical to the example PA, except for the designation which is varied to illustrate the modelled effect of different designations. The black bar shows the modelled r_d of the actual designation for the example PA. Vertical magenta line shows the observed r_d for the example PA. Only designations found in the country within which the example PA is located are modelled. These figures are for illustration of the observed variation among designations only, and do not imply that changing the designations of these reserves would result in different levels of protection.

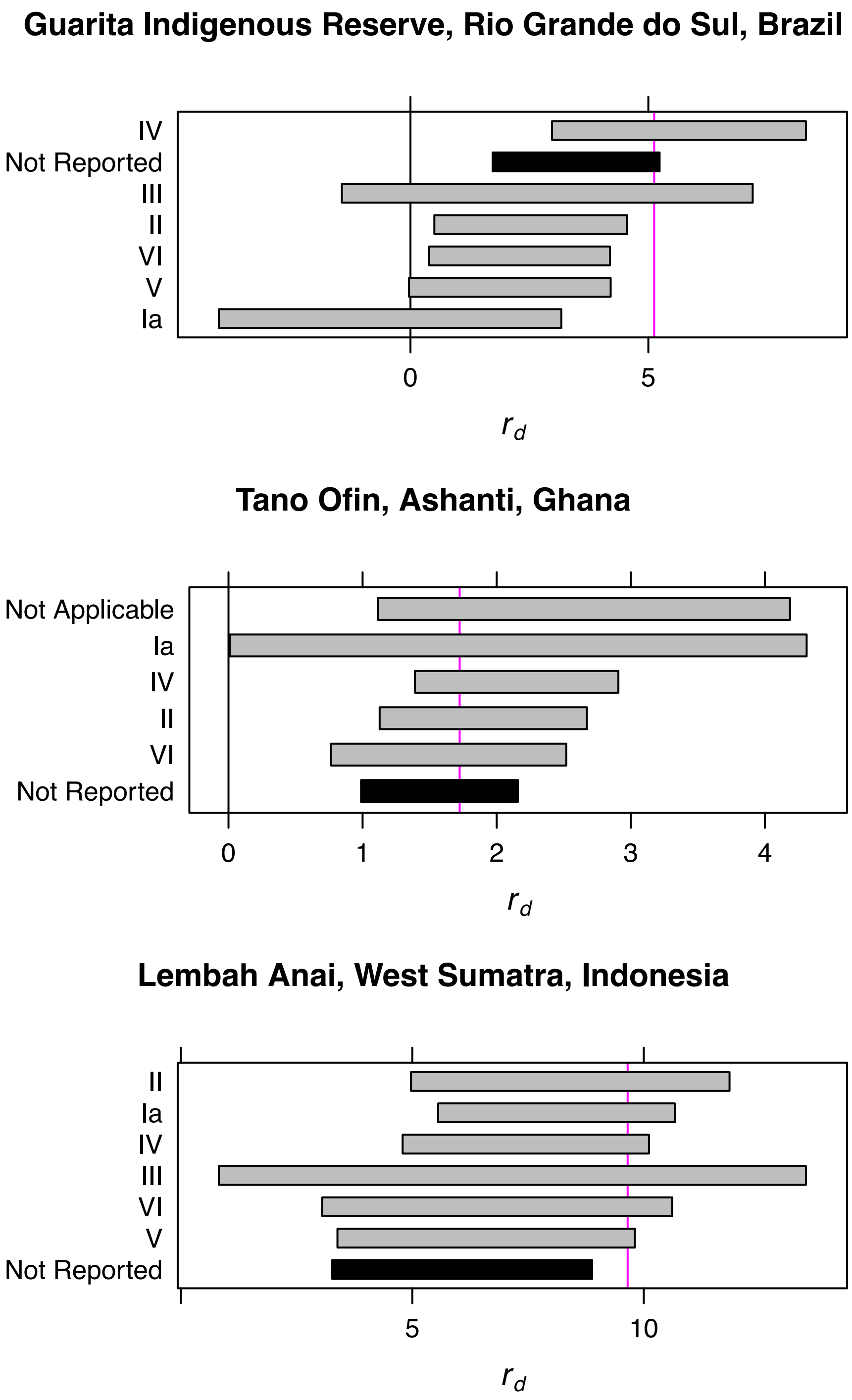

Fig. S5: Influence of IUCN category on r_d. Bars show 95 % Confidence Limits for predicted r_d for a hypothetical PA with properties identical to the example PA, except for the IUCN Category which is varied to illustrate the modelled effect of different designations. Details as in Figure S4 and Table S3.

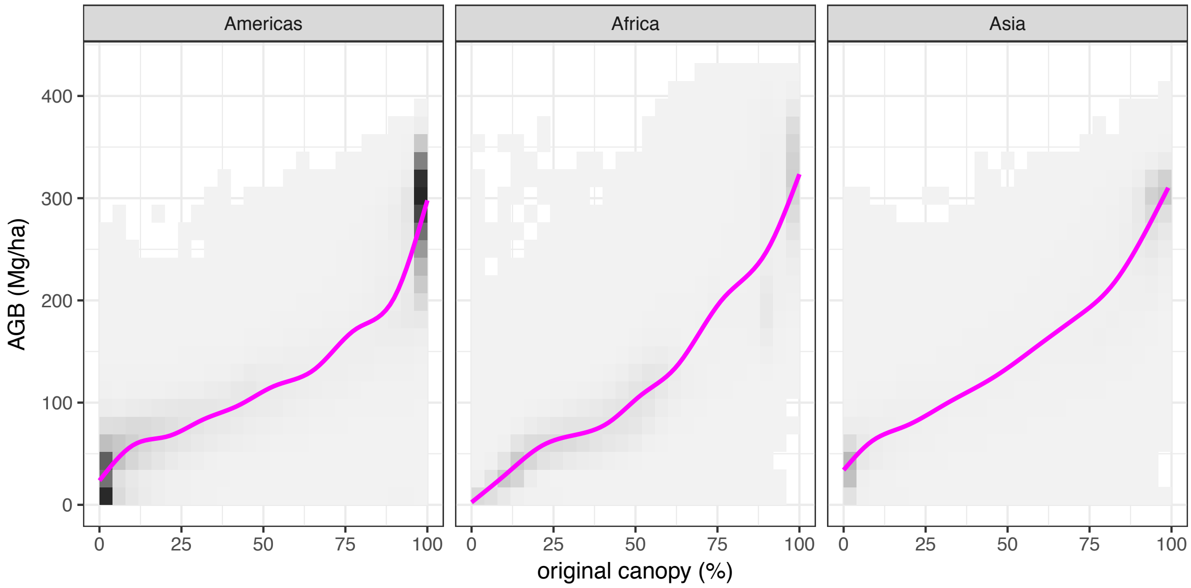

Fig. S6: Relationship between above ground biomass (AGB) and original canopy cover per 1 arc minute pixel, for those pixels that did not change canopy cover by more than 1 % between 2000 and 2012. Shading indicates data density, while magenta lines show cubic spline fits by GAM. R^2 was 92.7 % for the Americas, 90.0 % for Africa, and 90.8 % for Asia.

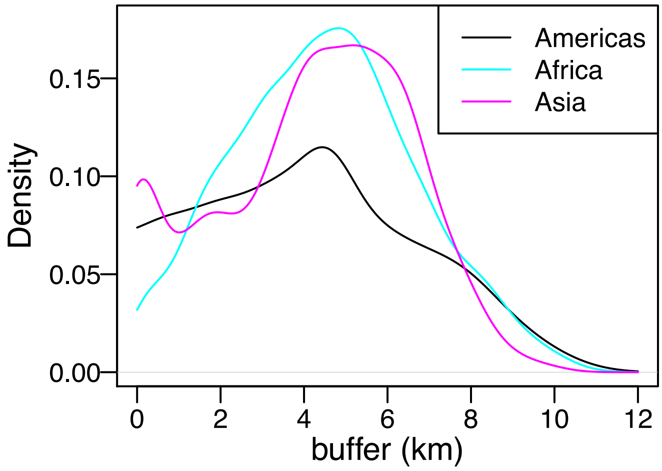

Fig. S7: Empirical probability density for buffer distances around PAs, used to test for the influence of leakage of deforestation from within to outside PAs. Buffer distance (km) was calculated as the natural logarithm of PA area (km2) to give values similar to those used in previous studies, but varying with PA area.

Figs. S8-S10 (below): Observed – predicted remaining AGB carbon for the Americas, Africa, and Asia, for the period 2000-2012. Polygons show PAs included in the study. Blue colours indicate more AGB remaining than expected by our models of forest loss in unprotected areas, red indicates less AGB remaining. Images were created using *R* v. 3.4.0. Specifically, rasters were created with package *raster* v.2.5.8, PA polygons were drawn using package *sp* v.1.2.4, and country polygons drawn using package *maps* v.3.2.0.

Fig. S8. Americas, observed – expected remaining AGB Carbon (Mg/ha)

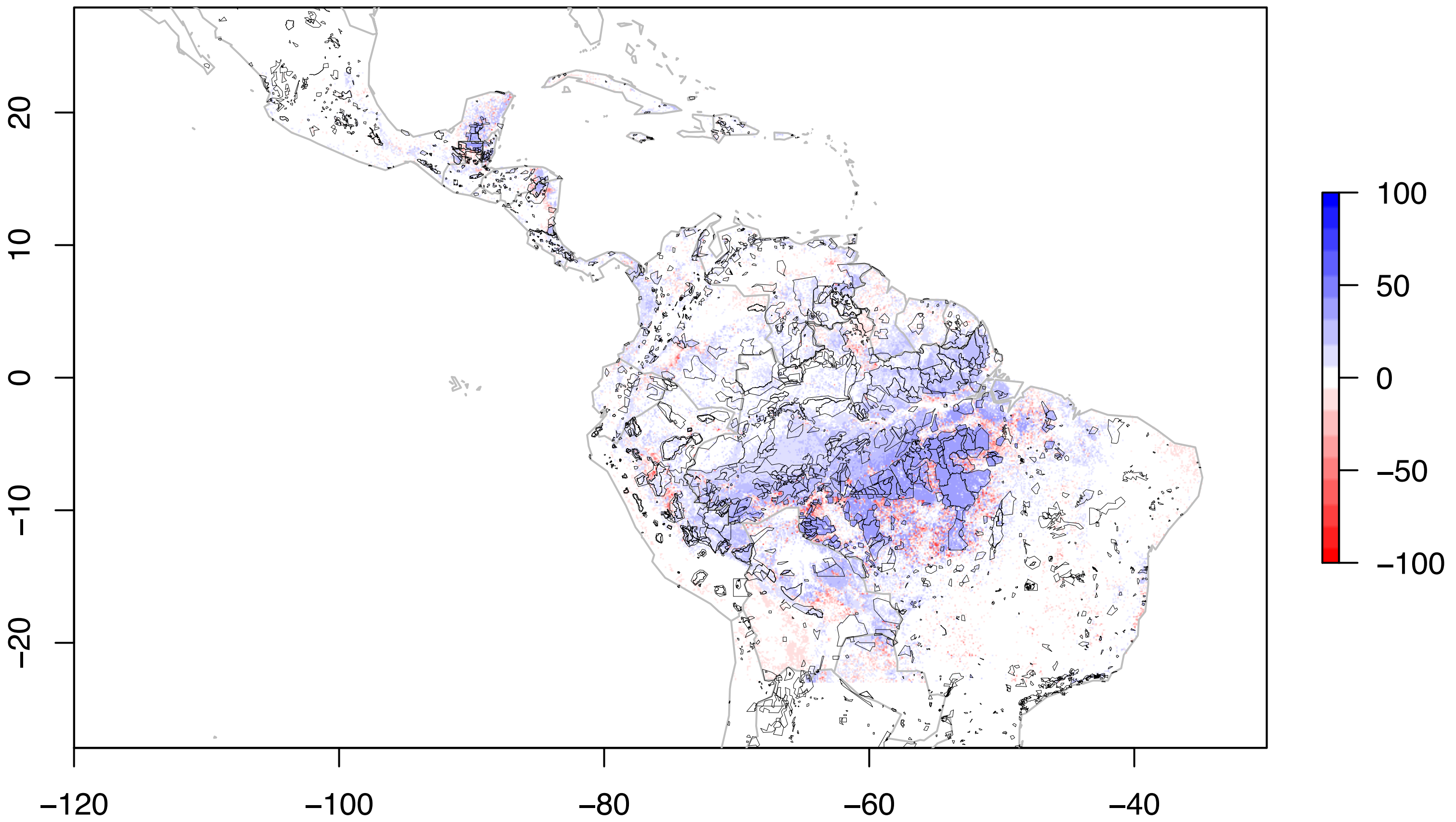

Fig. S9. Africa, observed – expected remaining AGB Carbon (Mg/ha)

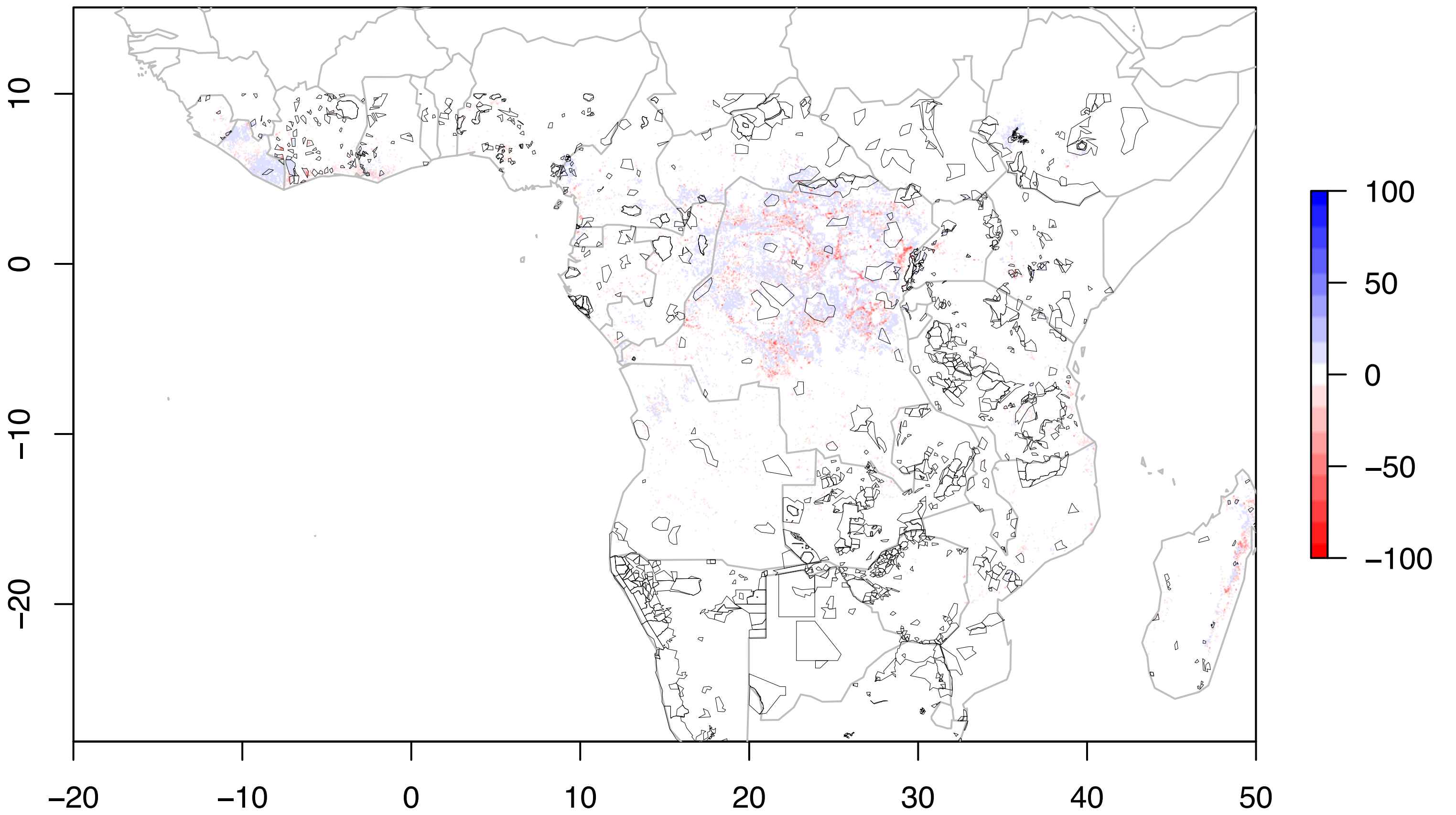

Fig. S10. Asia, observed – expected remaining AGB Carbon (Mg/ha)

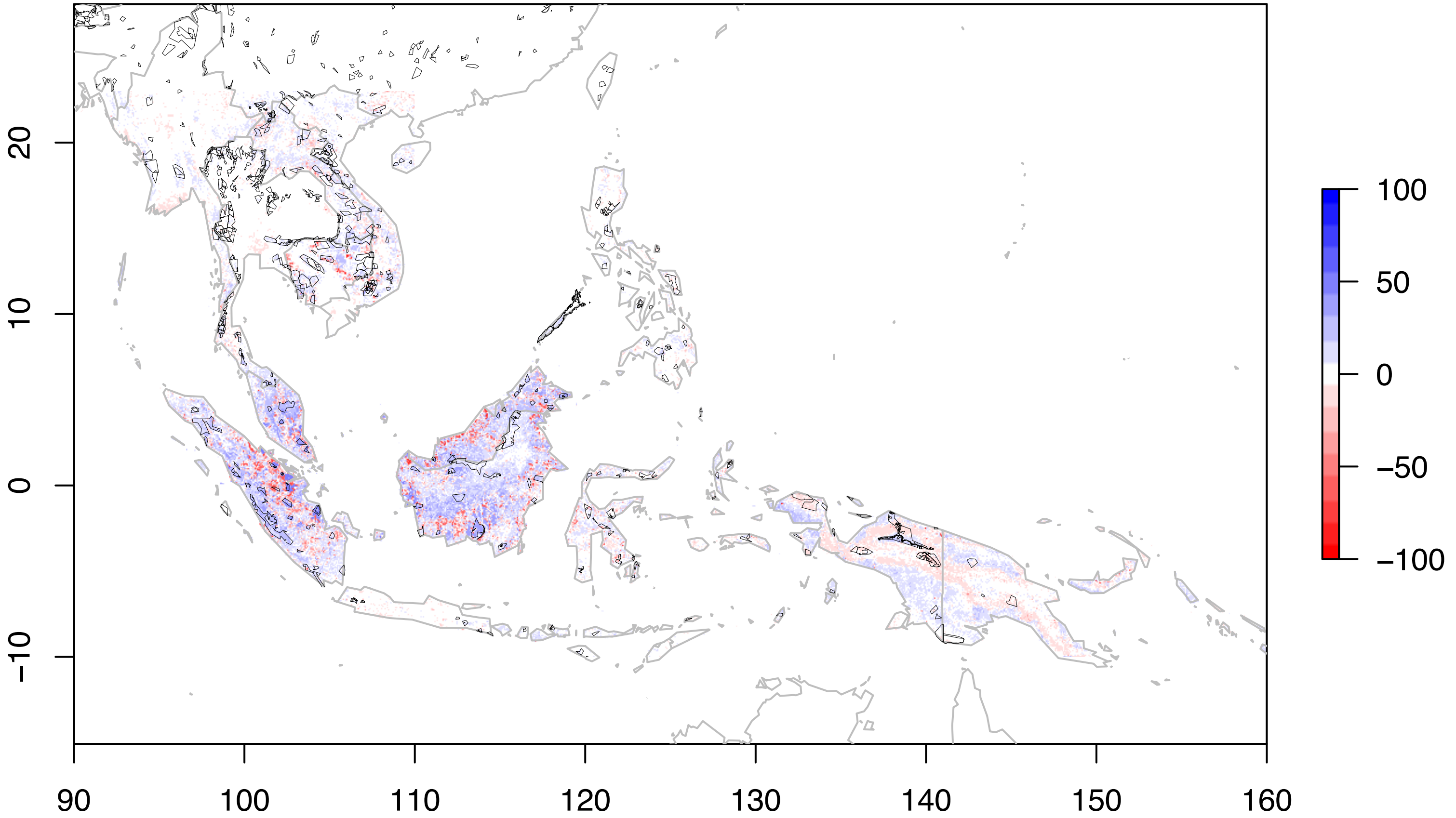

Tables

Table S1: Summary statistics of forest cover and PAs for the three tropical regions.

**Attribute**	**Americas**	**Africa**	**Asia**
Land surface area analysed (km^2)	17,929,928	15,316,162	6,403,597
Area with original canopy cover >20%	11,618,517	7,436,812	4,845,487
Area with original canopy cover >50%	8,649,980	3,604,886	3,800,909
PA area with canopy cover >20%	3,471,074	1,151518	619,659
PA area with canopy cover >20% in Tropics (below 23.4° latitude)	3,400,571	1,146,128	510,260
Non-PA area with canopy cover >20% in Tropics	7,180,988	4,992,355	3,441,244
Number of different PA designations	166	58	53
Number of PAs with original canopy cover >20%, area > 300 ha, designated by 2012	2409	1968	1049
Estimated annualized above ground biomass carbon loss (Pg C y^{-1}) in forested unprotected areas	0.39	0.14	0.26
Estimated annualized above ground biomass carbon loss (Pg C y^{-1}) in forested protected areas	0.068	0.013	0.015

Table S2: GAMs for r_d by PA. Categorical predictors were country, PA designation and IUCN category. Only PAs with original mean canopy cover greater than 20 % and area > 3 km^2 were included. Smooth terms were fitted using a tensor product smooth for spatial location (longitude and latitude), and cubic regression splines for original canopy cover, log area, the fraction of area covered by other PAs and designation year. Missing values in the designation year were replaced by the median. Subscripts below F denote numerator degrees of freedom. Degrees of freedom are estimated for smooth terms.

**Model data**	**Americas**	**Africa**	**Asia**
N (number of PAs)	2409	1968	1049
Residual DF	2166.6	1847.4	941.1
Spatial location	$F_{21.85}$ = 77.9, p < 0.001	$F_{14.6}$ = 5.7, p < 0.001	$F_{18.8}$ = 16.1, p < 0.001
Country	F_{33} = 9.5, p < 0.001	F_{27} = 5.5, p < 0.001	F_{15} = 2.3, p = 0.003
PA designation	F_{165} = 1.8, p < 0.001	F_{50} = 1.6, p = 0.004	F_{52} = 2.0, p < 0.001
IUCN category	F_{9} = 5.2, p < 0.001	F_{9} = 1.9, p = 0.06	F_{8} = 0.75, p = 0.65
Original canopy	$F_{5.2}$ = 29.9, p < 0.001	$F_{8.5}$ = 24.4, p < 0.001	$F_{3.0}$ = 6.5, p < 0.001
Log(area)	$F_{1.0}$ = 0.53, p = 0.46	$F_{3.2}$ = 1.75, p = 0.14	$F_{7.4}$ = 5.6, p < 0.001
Fraction single PA	$F_{7.9}$= 2.4, p = 0.009	$F_{8.3}$ = 4.06, p < 0.001	$F_{2.4}$ = 8.8, p = 0.001
Status year	$F_{3.8}$ = 6.8, p < 0.001	$F_{1.0}$ = 0.20, p = 0.66	$F_{3.9}$ = 1.7, p = 0.13
Deviance explained (%)	61.1	34.8	49.8

Table S3. Statistics for example PAs used in Figures S4 and S5.

Variable	Americas	Africa	Asia
Name	Guarita	Tano Ofin	Lembah Anai
Region	Rio Grande do Sul	Ashanti	West Sumatra
Country	Brazil	Ghana	Indonesia
Coordinates	53.6°W, 27.44°S	2.15°W, 6.71°N	100.5°E, 0.86°S
Area (ha)	23399	40223	95438*
Established	1994	1929	1922
Designation	Indigenous Area	Classified Forest Reserve	Nature Reserve
IUCN Category	Not reported	Not reported	Not reported
Original canopy (%)	69.0	69.0	94.5
Remaining canopy (%)	68.3	67.6	93.9
Predicted remaining (%)	65.0	64.7	87.3

*Lembah Anai is reported as being 221 ha but this does not accord with the polygon in the WDPA. We used areas of polygons rather than reported areas in our analyses.

Table S4. Summary data by country. Only tropical forest areas (between 23.4°N and 23.4°S) with mean original forest cover greater than 20 per cent (at 1 arc minute resolution) are included in the analysis. Forest Area is the original area of forest with mean cover greater than 20 per cent. PA Area is the total area under protection with original cover greater than 20 per cent. PA Fraction is the PA area as a fraction of the Forest Area. Expected change is the mean change in forest cover within PAs predicted by the GAMs. Actual change is the observed change in forest cover within PAs. Carbon saved is in Mg per year averaged over the 12 years of the analysis. The data are ordered by Forest Area within Region, and only countries with more than 1000 km^2 original forest cover are listed. Variation in carbon density means that carbon can be lost even if forest cover loss is less than expected, and vice versa. Tg is equivalent to Mt.

**AMERICAS**						
**Country**	**Forest Area (km^2)**	**PA Area (km^2)**	**PA Fraction**	**Expected change (%)**	**Actual change (%)**	**Carbon saved ($Tg\ y^{-1}$)**
Brazil	5591447	2102679	0.38	-6.00	-1.05	301.6
Colombia	919069	142951	0.16	-3.35	-1	5.72
Peru	801805	308821	0.39	-3.03	-1.28	16.5
Bolivia	701848	179868	0.26	-4.20	-1.17	12.7
Venezuela	639533	331139	0.52	-3.24	-0.73	13.7
Mexico	511432	64858	0.13	-3.92	-0.91	4.68
Ecuador	208877	37164	0.18	-1.24	-0.4	1.12
Guyana	196746	18415	0.09	-0.69	-0.11	0.56
Paraguay	189790	13665	0.07	-8.86	-2.16	0.88
Suriname	141809	20236	0.14	0.19	-0.16	0.16
Nicaragua	101296	32781	0.32	-6.64	-8.15	1.49
Honduras	94852	20165	0.21	-4.75	-5.95	0.32
Guatemala	92975	32878	0.35	-7.88	-10.87	4.40
French Guiana	83211	36765	0.44	-1.93	-0.22	2.37
Panama	65808	14583	0.22	-3.93	-0.93	1.35
Cuba	51082	3680	0.07	-4.87	-2.11	0.25
Costa Rica	48238	11595	0.24	-3.46	-0.83	0.89
Dominican Republic	34470	7709	0.22	-5.13	-3.92	0.24
Argentina	33637	7506	0.22	-6.00	-2.45	0.00
Belize	19782	7663	0.39	-7.29	-2.42	1.23
El Salvador	14367	1408	0.1	-2.65	-1.85	0.06
Jamaica	9557	1460	0.15	-3.81	-2.57	0.08
Puerto Rico	7290	458	0.06	-3.93	-0.92	0.03
Trinidad and Tobago	4274	1483	0.35	-4.90	-3.1	0.14
Guadeloupe	1261	10	0.01	19.1	-0.22	0.00
Martinique	979	30	0.03	5.18	-0.77	0.02

AFRICA						
Country	Forest Area (km^2)	PA Area (km^2)	PA Fraction	Expected change (%)	Actual change (%)	Carbon saved (Tg y^{-1})
Democratic Republic of the Congo	2251850	234394	0.10	-1.75	-0.82	5.59
Angola	756807	17302	0.02	-1.27	-1.07	0.01
Central African Republic	559441	87681	0.16	-0.58	-0.25	0.70
United Republic	446384	168535	0.38	-1.10	-0.67	0.84
Mozambique	426655	63403	0.15	-0.95	-0.52	0.29
Zambia	405278	146105	0.36	-0.72	-0.61	0.26
Cameroon	378707	42481	0.11	-1.73	-0.26	1.14
Congo	321612	35256	0.11	-0.31	-0.29	0.16
Gabon	230833	28971	0.13	-1.02	-0.25	0.42
Sudan	225429	39585	0.18	-0.75	-0.08	0.21
Cote d'Ivoire	224785	53401	0.24	-2.53	-4.10	-0.38
Madagascar	190140	30396	0.16	-2.93	-2.86	0.42
Nigeria	170321	33386	0.20	-0.93	-1.66	-0.08
Ethiopia	163092	56906	0.35	-2.03	-0.74	1.08
Uganda	134831	21557	0.16	-2.29	-1.56	0.27
Liberia	95594	11521	0.12	-5.07	-0.63	0.91
Ghana	94494	19168	0.20	-2.44	-1.91	0.30
Guinea	71175	4030	0.06	-2.31	-0.63	0.11
Sierra Leone	68878	3997	0.06	-1.93	-2.29	0.02
Kenya	55573	14887	0.27	-2.59	-2.17	0.21
Malawi	30695	13258	0.43	-0.42	-0.89	-0.04
Equatorial	26766	5071	0.19	-2.17	-0.55	0.15
Zimbabwe	20475	4785	0.23	-1.32	-1.50	-0.02

ASIA						
**Country**	**Forest Area (km^2)**	**PA Area (km^{2})**	**PA Fraction**	**Expected change (%)**	**Actual change (%)**	**Carbon saved ($Tg\ y^{-1}$)**
Indonesia	1724296	166957	0.10	-5.28	-2.09	11.7
Papua New Guinea	438638	12642	0.03	0.99	-0.65	-0.24
Burma	322531	28049	0.09	-2.72	-1.21	0.77
Malaysia	318881	32065	0.10	-10.46	-2.72	5.49
Thailand	229583	99999	0.44	-2.01	-0.97	2.09
Philippines	220349	31313	0.14	-2.33	-2.02	0.40
Lao People's Democratic Republic	209992	43071	0.21	-4.95	-2.37	2.20
Viet Nam	200378	39603	0.20	-4.57	-3.25	1.35
China	117090	12305	0.11	-6.00	-3.31	0.43
Cambodia	106894	40584	0.38	-6.03	-5.77	0.90
India	18969	603	0.038	-5.31	-3.76	0.01
Bangladesh	13559	885	0.07	-3.90	-3.40	0.00
Timor-Leste	10417	1010	0.10	-2.06	-1.07	0.02
Brunei Darussalam	5607	1139	0.20	0.25	-1.30	-0.04

Data sources and processing

All data manipulation was carried out in the R v. 3.4.0 (downloaded from http://cran.r-project.org). Where required, additional processing of ESRI Shapefiles was conducted in QGIS (http://www.qgis.org).

World Database on Protected Areas (WDPA)
Reference: IUCN, UNEP-WCMC 2015. The World Database on Protected Areas (WDPA). July 2015. Cambridge (UK): UNEP World Conservation Monitoring Centre.
Available from: http://www.protectedplanet.net
Download date: July 2015.
Description from source: The World Database on Protected Areas (WDPA) is a joint project between the United Nations Environment Programme (UNEP) and the International Union for Conservation of Nature (IUCN), managed by UNEP World Conservation Monitoring Centre (UNEP-WCMC). The dataset described here shows the global distribution of terrestrial and marine protected areas as well as sites that do not meet the standard definition of a protected area but do achieve conservation in the long-term, generically referred to as other effective area-based conservation measures (OECMs). Throughout this metadata document, protected areas and OECMs are collectively referred to as conservation areas. The dataset contains protected areas designated at the national level and under regional and international conventions and agreements. International designations include those under the Ramsar Convention, the World Heritage Convention (United Nations Educational, Scientific and Cultural Organization, UNESCO), and sites under the UNESCO's Man and the Biosphere Programme (MAB). Regional agreements include sites under the Natura 2000 network (European), as well as Marine Protected Areas designated under regional conventions such as the Convention for the Protection of the marine Environment of the North-East Atlantic (OSPAR) and many others. It also contains data on protected areas and OECMs established by other means.
Format: ESRI Shapefile polygons, WGS84 coordinate reference system.
Processing: The WDPA database was imported to R using the *readOGR* function (*rgdal* library). We checked the validities of polygon geometries by applying the *gIsValid* function (*rgeos* library) to each polygon in turn. The WDPA polygons contained numerous topological errors (e.g. orphaned holes, self-intersections). We located the errors using the output of *gIsValid*, imported the WDPA database into QGIS Geographic Information System software, and manually edited the shapefile to remove the errors. Once all errors were corrected, we simplified the geometry in QGIS to a tolerance of 0.001° (approx. 110m at the equator) to facilitate manipulation of the data. All PAs were retained which were designated by the year 2012, had a mean original canopy cover of >20 %, and for which a shapefile was available.

Global Forest Change
Reference: Hansen, M. C. et al. High-Resolution Global Maps of 21st-Century Forest Cover Change. Science 342, 850–853 (2013).
Available from: https://earthenginepartners.appspot.com/science-2013-global-forest
Download date: March 2014
Description from source: Results from time-series analysis of Landsat images characterizing forest extent and change. Trees are defined as vegetation taller than 5m in height and are expressed as a percentage per output grid cell as ‘2000 Percent Tree Cover’. ‘Forest Cover Loss’ is defined as a stand-replacement disturbance, or a change from a forest to non-forest state, during the period 2000–2012. ‘Forest Cover Gain’ is defined as the inverse of loss, or a non-forest to forest change entirely within the period 2000–2012. ‘Forest Loss Year’ is a disaggregation of total ‘Forest Loss’ to annual time scales. Reference 2000 and 2012 imagery are median observations from a set of quality assessment-passed growing season observations.
Format: GeoTIFF tiles covering 10 x 10° regions globally at 1 arc-second resolution (approx. 30m at the equator), WGS84 coordinate reference system.

Processing: We downloaded tiles covering the major tropical forest regions, for original forest cover in 2000 and loss of cover by 2012. The spatial extent 30°W–120°W and 30°S–30°N for the Americas, 20°W–50°E and 23°S–10°N in Africa, and 90°E - 160°E and 10°S–30°N in Asia. We imported the data into R using the *raster* function (*raster* library). All raster image analyses were conducted using functions in the *raster* library. We merged tiles using the *merge* function to give single rasters for each region. We calculated remaining forest cover by converting original forest cover to zero in pixels identified as having lost forest cover by 2012. We aggregated original forest cover and remaining forest cover to means at 1 arc-minute resolution (approx. 1.8km resolution at the equator) using the *aggregate* function, to facilitate calculations. This resulted in 4,601,128 land surface pixels in Africa, 5,449,272 in the Americas, and 1,957,368 in Asia.

World Borders Dataset
Reference: Sandvik, B. 2009. TM_WORLD_BORDERS-0.3. Thematic Mapping.
Available from: http://thematicmapping.org/downloads/world_borders.php
Download date: March 2014
Description: World national borders polygons.
Format: ESRI Shapefile polygons, WGS84 coordinate reference system.
Processing: We imported this dataset into R using function *readOGR* and used it without modification.

Gridded Population of the World Version 3 (GPWv3)
Reference: Center for International Earth Science Information Network (CIESIN), Columbia University; and Centro Internacional de Agricultura Tropical (CIAT). 2005.
Gridded Population of the World Version 3 (GPWv3): Population Density Grids. Palisades, NY: Socioeconomic Data and Applications Center (SEDAC), Columbia University.
Available from: http://sedac.ciesin.columbia.edu/gpw
Download date: March 2014
Description from source: This archive contains population densities, both UN-adjusted and unadjusted. The raster data are at 2.5 arc-minutes resolution and contain the following data:
ds00g population densities in 2000, unadjusted, persons per square km
ds00ag population densities in 2000, adjusted to match UN totals, persons per square km
The data are stored in geographic coordinates of decimal degrees based on the World Geodetic System spheroid of 1984 (WGS84).
Format: ASCII grid at 2.5 arc minute resolution, WGS84 coordinate reference system. **Processing**: We used the unadjusted population density in the year 2000. We disaggregated the data to 1 arc-minute resolution using the *disaggregate* function (*raster* library) in R, with bilinear interpolation. The distribution of values was highly skewed, therefore we transformed the data as $\log(x + 1)$ for inclusion as a predictor.

Global Roads Inventory Project (GRIP) Version 3
Reference: PBL Netherlands Environmental Assessment Agency. 2013. Global Roads Inventory Project (GRIP) version 3. The Hague, Netherlands.
Available from: https://data.overheid.nl/data/dataset/grip-v3-2013-densities-global-roads- inventory-project
Download date: March 2014
Description from source: The road density raster datasets are derived from the Global Roads Inventory Project vector dataset. The raster datasets contain the kilometers of road per 5 arc- minute grid cell and are available for the total amount (all roads equal) and also per road type: highways/motorways (1), primary roads (2), secondary roads (3), tertiary roads (4) and local/urban roads (5). The current road density raster is based on GRIP version 3 (2013). The Global Roads Inventory Project (GRIP) database is developed by PBL. The GRIP database was created in order to provide a more recent and accurate global roads database compared to the well-known but outdated DCW/VMAP datasets. GRIP is used in PBL's global environmental assessment activities, but also by other institutes and organizations around the world. GRIP is based on data that was collected from many (50+) mainly publicly available sources, for instance National Spatial Data

Infrastructures, topographic agencies, NGOs, Universities, UN agencies, etc. Also parts of GRIP are derived from data created by the OpenStreetMap project (www.openstreetmap.org). To create a consistent and transparent global roads database that can be useful for various types of analyses and also be maintained with help of a larger community the United Nations UNSDI-Transportation datamodel ("v2 light") was used. This datamodel was developed by the Logistics Cluster of the UN World Food Programme. More information on UNSDI-T can be found at http://www.logcluster.org/tools/mapcentre/unsdi/unsdi-t-v2.0/ The ambition of GRIP is to create a dataset that is: globally consistent (in terms of the underlying data model and attribute coding); spatially accurate (~50-100m positional accuracy); topologically integrated; suitable for mapping at an approximate scale of 1:100,000 - 1:1000,000; focused on roads between settlements (not streets); up-to-date and with the possibility of frequent updates; preferably well documented; publicly available (on an "attribution only" basis)
Format: ASCII grid at 5 arc minute resolution, WGS84 coordinate reference system. **Processing**: We imported the data into R and disaggregated to 1 arc-minute resolution. The distribution of values was highly skewed, therefore we transformed the data as $\log(x + 1)$ for inclusion as a predictor.

Agricultural Suitability

Reference: Zabel, F., Putzenlechner, B. & Mauser, W. Global Agricultural Land Resources – A High Resolution Suitability Evaluation and Its Perspectives until 2100 under Climate Change Conditions. PLoS ONE 9, e107522 (2014).
Available from: http://geoportal-glues.ufz.de/stories/globalsuitability.html
Download date: July 2014
Description from source: General agricultural suitability at a spatial resolution of 30 arc seconds, considering rainfed conditions and irrigation on currently irrigated areas. The agricultural suitability represents for each pixel the maximum suitability value of the considered 16 plants. The dataset contains four time periods (1961-1990, 1981-2010, 2011-2040, 2071-2100). Local climate, soil and topography determine the available energy, water and nutrient supply for agricultural crops and thus their natural suitability. In order to allow for computing the natural agricultural constraints on the globe at 30 arc seconds spatial resolution, the following high resolution data were applied: Daily data for temperature, precipitation and solar radiation from the global climate model ECHAM5. Soil data comes from the Harmonized World Soil Database (HWSD). Considered soil properties are texture, proportion of coarse fragments and gypsum, base saturation, pH content, organic carbon content, salinity, sodicity. Topography data was applied from the Shuttle Radar Topography Mission (SRTM). Irrigation has strong impact on the crop's suitability. It is considered on todays irrigated areas as given by the FAO Aquastat Global Maps of Irrigated Areas (GMIA) dataset. The determinant factors are contrasted with the crop-specific requirements, using a fuzzy-logic approach. The crop requirements are taken from literature.
Format: ASCII grid at 30 arc second resolution, WGS84 coordinate reference system.
Processing: We imported the 1981-2010 estimates, and aggregated these to 1 arc minute resolution.

Global Terrain Slope and Aspect Data

Reference: Fischer, G., F. Nachtergaele, S. Prieler, H.T. van Velthuizen, L. Verelst, D. Wiberg, 2008. *Global Agro-ecological Zones Assessment for Agriculture (GAEZ 2008)*. IIASA, Laxenburg, Austria and FAO, Rome, Italy.
Available from: http://webarchive.iiasa.ac.at/Research/LUC/External-World-soil-database/HTML/global-terrain-slope-download.html?sb=7
Download date: March 2014
Description from source: The global terrain altitude, slope and aspect database has been compiled using elevation data from the Shuttle Radar Topography Mission (SRTM). The SRTM data is publicly available as 3 arc second (approximately 90 meters resolution at the equator) DEMs. The high resolution SRTM data have been used for calculating: Median altitude; Terrain slope gradients and classes for each 3 arc-sec grid cell; Aspect of terrain slopes for each 3 arc-sec grid cell; Distributions of slope gradient and slope aspect classes for 30 arc second grid.
A global terrain slope and aspect database comprises the following elements: Elevation (median)

Slope gradient: Distributions of nine slope gradient classes are available for each grid-cell: 0–0.5%, 0.5–2%, 2–5%, 5–8%, 8–16%, 16–30%, 30–45%, and > 45%.
Slope aspects: Slope aspect data is stored in distributions of five classes namely: Class 1: slopes below 2% undefined aspect; Class 2: slopes facing North (315°–45°); Class 3: East (45°–135°); Class 4: South (135°–225°), and Class 5: West (225°–315°).
Format: ASCII grid at 30 arc second resolution, WGS84 coordinate reference system.
Processing: We imported the grids for altitude, and the fraction of slope gradient classes 16–30%, 30–45%, and > 45%, merging the three classes to give a single raster for the fraction of a pixel with gradient above 15°. We then aggregated these to the mean at 1 arc minute resolution. The distribution of values was highly skewed, therefore we transformed the data as $\log(x + 1)$ for inclusion as a predictor.

Major World Ecosystem Complexes Ranked by Carbon in Live Vegetation: A Database
Reference: Olson, J.S., J.A. Watts, and L.J. Allison, 1985. Major World Ecosystem Complexes Ranked by Carbon in Live Vegetation (NDP-017). Carbon Dioxide Information Center, Oak Ridge National Laboratory, Oak Ridge TN.
Available from: http://cdiac.ornl.gov/ndps/ndp017.html
Download date: December 2015
Description from source: In 1980, this database was completed after more than 20 years of field investigations, consultations, and analyses of published literature. The data characterize the use and vegetative cover of the Earth's land surface with a 0.5° × 0.5° grid. This world- ecosystem-complex data set and the accompanying map provide a current reference base for interpreting the role of vegetation in the global cycling of CO_2 and other gases and a basis for improved estimates of vegetation and soil carbon, of natural exchanges of CO_2, and of net historic shifts of carbon between the biosphere and the atmosphere.
Format: ASCII grid at 0.5° resolution, WGS84 coordinate reference system
Processing: We imported the dataset to R and disaggregated the ecosystem classes to 1 arc minute resolution. This dataset was not included in the final model relating forest cover to biomass (see Methods).

Pantropical Aboveground Biomass
Reference: Baccini, A. *et al.* Estimated carbon dioxide emissions from tropical deforestation improved by carbon-density maps. *Nature Clim. Change* 2, 182–185 (2012).
Available from: http://whrc.org/publications-data/datasets/pantropical-national-level-carbon-stock/
Download date: October 2014
Description from source: Woods Hole Research Center scientists and their collaborators generated a national level aboveground dataset for tropical countries. Using a combination of co-located field measurements, LiDAR observations and imagery recorded from the Moderate Resolution Imaging Spectroradiometer (MODIS), WHRC researchers produced national level maps showing the amount and spatial distribution of aboveground carbon. Units are Mg/ha. Data are supplied in three tiles, for the Americas, Africa, and Asia. Latitudinal extent is limited to the tropics *sensu stricto* 23.4°S to 23.4°N.
Format: GeoTIFF grid at 463.3 m resolution, sinusoidal coordinate reference system.
Processing: We imported the grids to R, and reprojected them to match the forest cover data at 1 arc minute resolution using the *projectRaster* function (*raster* library). Aboveground biomass was converted to carbon by assuming a conversion of 0.5.
